# A Letter to the Commissioners for Transports and Sick and Wounded Seamen, on the Non-Contagious Nature of the Yellow Fever; and, Containing Hints to Officers for the Prevention of This Disease among Seamen

**Published:** 1818-05

**Authors:** 


*A Letter to the Commissioners for Transports and sick and wounded Seamen, on the Non-contagious Nature of the Yellow Fever ; and, containing Hints to Officers for the Prevention of this Disease among Seamen.* By JAMES VEITCH, M. D. Staff Surgeon to the Navy, &c. &c. London, 1818, pp. 173.

HOWEVER erroneous may be the opinions of Dr. Chisholm and Dr. Pym respecting the "Bulam Fever," their publication has done much good, both in promoting a liberal discussion amongst the profession, (than which nothing tends more to the advancement of knowledge) and in causing publicity to be given to a number of valuable observations on the diseases of tropical climates, which, perhaps, without the *impulse* of controversy, might have remained in obscurity.

The "Letter" commences with several extracts from the "African Memoranda," on which Dr. Veitch makes some judicious comments. The transition from the temperature of 32°, that of the winter in England, to 135°, that of the climate of Bulam, at the arrival of the colonists, must have proved highly injurious to their unseasoned constitutions; and this, together with the exertion of clearing the grounds in an atmosphere loaded with miasmata, is sufficient to account for the production of fevers, without calling in the aid of contagion. These appear to have made an early impression on the colony; and that they should prove destructive to people so circumstanced, without medical aid, is not surprising, especially when we consider the indiscriminate use of emetics, bark, and wine, by their humane, but in this instance mistaken leader Captain Beaver. Such is the irritability of the stomach in yellow fever, that these medicines would not only have been instantly rejected, but have killed the patient in less than twenty-four hours,—and yet we see some of the colonists lived for more than a month under such treatment; a striking proof that the fever they suffered from was not the Yellow Fever. Besides, we find that all ages and sexes were attacked; that Mr. Beaver himself, while at Bulam, had six different seizures, and it appears from his list, that the complaint was intermittent; all which circumstances militate against its being the Yellow Fever.

That the fever which afflicted the colonists was not contagious, we may conclude from the following circumstances. They visited Sierra Leone and other settlements without communicating it to any one; the Calypso had it on board for nearly a month, with the loss of only one; during the stay of the Hankey at Bulam, she did not lose one of her crew. The fever was not the offspring of filth, for Capt. Beaver paid strict attention to cleanliness and ventilation.

"Such a chain of events," continues Dr. V. "are incompatible with a contagious disease; and there is just cause for concluding, that no such fever existed among the colonists, as that ascribed to them by Dr. Chisholm and Dr. Pym, under the head of Bulam Fever."

Having finished his observations on the "African Memoranda", Dr. Veitch proceeds to make a few extracts from the work of Dr. Chisholm, "the parent and propagator of the Bulam Fever." We must pass these over, selecting what we think most worthy from our author's "Remarks" on them.

The atmosphere of the West Indies is unfriendly to the generation and propagation of infectious diseases; and had accumulated filth, anxiety, and want of ventilation been capable of producing pestilential fever between the tropics, the crews of those ships which encountered Rodney would have been extirpated, as they were proverbially dirty. They were, however, more healthy than their cleanly opponents, the English.

The state of slave ships is another proof of the same doctrine, in which climate obviates the generation of contagion under circumstances which must have given origin to it, but for the non-existence of predisposition amongst the negroes, and the hostile tendency of the torrid zone to propagate such causes of destruction. Were filth in the extreme capable of generating contagion, there is scarcely a negro hut in the West Indies, that would not have become a focus of contagion.

Dr. Chisholm's statements that,—the colony at Bulam was destitute of good water; that the colonists were obliged to live on board the Hankey during nine months; and that the bedding, &c. of those who died were not destroyed, are erroneous. Nor did the Hankey ever return to Bulam, or take men on board from Sierra Leone.

The disease described by Dr. C. is not a new disease. It was known in 1726, and may be considered as coeval with the attempt of Europeans to settle in tropical climates. It is evident, even from the Doctor's own account, that the causes of the fever he described were local, and independent of imported contagion—thus we see the persons attacked, were from the age of 15 to 50, robust and vigorous, recently arrived from Europe; had bathed whilst intoxicated, exposed to the sun; had slept on deck, and indulged in new rum. These were sufficient to produce fever in any climate. It is singular that it should be ascribed to imported contagion from Bulam.

The ship Hankey arrived at Grenada on the 19th of February, and it was not until the middle of April that the fever got ashore. Can it be supposed that had this ship been so virulently contagious, it would not have visited the shore, with which there was free intercourse, at an earlier period? Mr. Smithers, surgeon of the Charon, was examined before the Governor, and gave his opinion, that the disease did not originate from the Hankey. This circumstance is not mentioned by Dr. C.

The Bulam Fever of Dr. Pym might, with much more propriety, have been called the Grenada, Curaçoa, or Barbadoes Fever, for in all these islands it had shewn itself before 1793.

Patients labouring under synocha, pleurisy, gastritis, (which has a strong analogy to Bulam or Yellow Fever) hepatitis, enteritis, or any other inflammatory disease; particularly where the cure has been decided by well timed evacuations, are by no means disposed to an immediate recurrence of these maladies in their active form; but they are not secured from subsequent attacks (which will be found milder) of those diseases on their recovering their full vigour, and being again exposed to their causes; "and such precisely," says Dr. Veitch, "is my opinion of yellow fever. The restoration of vigour, and of susceptibility by returning to Europe, or even going to sea or the West Indies, will, on exposure to the causes, reproduce the disease."

Boys under puberty, men advanced in life, and women are comparatively exempted from yellow fever; which circumstances arise from the state of the constitution, as well as habits of life. An attack of dysentery, remittent or intermittent fever, will as effectually secure the patient as if he had suffered from the disease, by lowering the system.

Epidemical diseases are too often confounded with contagious ones. The invasion of catarrhal affection is a proof of the rapidity of climate in diffusion of disease. We cannot refrain from giving the following extract in the words of our author.

"Let us suppose the inhabitants of a tropical country, to the number of 275, men, women and children, landed in Scotland for the purpose of colonization, at the most unfavourable season of the year; and that they proceed immediately to the object of their destination; can there be a doubt as to the consequences; and that climate would very shortly prove fatal to the greater number of them? Catarrh, diarrhœa, dysentery, pleurisy, frost-bitten limbs, would soon diminish their numbers. And, if this view of the influence of our own climate be just, assuredly the converse mode of reasoning cannot be rejected. Their countrymen, on hearing the fate of their friends, would entertain a frightful idea of our climate, and contagion would probably heighten the picture; quarrantine laws would be thought of; and if they possessed the power of acting on such suggestions, we, as the inhabitants of Great Britain, would certainly not admit their precautions to be founded in truth or necessity."

In the treatment f yellow fever, blood-letting "holds the highest place":—Convalescence is rapid, the patients emerge from disease almost at once into health, so that they seldom require tonics; when these are necessary, quassia is prefered to bark. On dissection, the brain exhibits marks of increased action. The stomach and small intestines, the liver, gall-bladder, the heart and great vessels, the lungs, all partook of the same appearances.

The Letter concludes with a long quotation from the author's Treatise, "De methodo tractandi et peccavendi febrem flavam," published in 1818; and an Appendix is added, containing several extracts from the works of Rush, Bancroft, and others, on the subject of Yellow Fever.

Dr. Veitch is a man of considerable abilities and ample experience in the diseases of tropical climates; the present letter is worthy of him, and must be considered as no small addition or trifling auxiliary on the side of the "Non-contagionists."

---

*Observations on the Harveian Doctrine of the Circulation of the Blood.* By George Kerr. Small 8vo. pp. 100. London, 1816.

